# Recurrence of macular edema in patients with branch retinal vein occlusion: a proteomic study

**DOI:** 10.1186/s12886-024-03359-z

**Published:** 2024-02-22

**Authors:** Yin Liu, Xiaohu Wang, Yonghong Sheng, Haili Jin, Linfeng Han, Jun Xu, Qingqing Fu, Jing Liu, Feng Ji, He Ding, Xiaochen Xu, KunChao Wu, Pengfei Zhang, Guoping Wang

**Affiliations:** 1 Wuhu Eye Hospital, No. 378, Santan Road, Yijiang District, Wuhu, Anhui Province 241000 China; 2 Department of Ophthalmology, First People’s Hospital of Guiyang, Guiyang, China; 3 Department of Ophthalmology, The First Affiliated Hospital of Wannan Medical College (Yijishan Hospital of Wannan Medical College), Wuhu, China

**Keywords:** Branch retinal vein occlusion, Macular edema, Aqueous humor, Proteomics, Complement and coagulation cascades, Platelet activation

## Abstract

**Background:**

Branch retinal vein occlusion (BRVO) is a common retinal vascular disease leading to severe vision loss and blindness. This study aimed to investigate and reveal the pathophysiological mechanisms underlying macular edema (ME) recurrence in patients with BRVO through a proteomic approach.

**Methods:**

We detected proteins in the aqueous humor of 14 untreated, four refractory, and four post-operative patients with BRVO-ME and 12 age-matched cataract controls using four-dimensional label-free proteomic and bioinformatics analyses.

**Results:**

In total, 84 proteins exhibited significant differential expression between the BRVO and control samples (fold change [FC] ≥ 1.2 and adjusted *p*-value < 0.05). Compared to the control group, 43 and 41 proteins were upregulated and downregulated, respectively, in the BRVO group. These proteins were involved in cell adhesion, visual perception, retina homeostasis, and platelet activation. Several significantly enriched signaling pathways included complement and coagulation cascades and platelet activation. In the protein–protein interaction networks generated using the search tool for retrieval of interacting genes (STRING), the fibrinogen alpha chain and fibrinogen beta chain constituted a tightly connected cluster. Many common protein expression trends, such as the fibrinogen alpha chain and fibrinogen beta chain, were observed in both the recurrent and refractory groups. Differentially expressed proteins in the two groups were involved in complement activation, acute-phase response, platelet activation, and platelet aggregation. Important signaling pathways include the complement and coagulation cascades, and platelet activation. Protein–protein interaction analysis suggested that the fibrinogen alpha chain and fibrinogen beta chain constituted a tightly connected cluster. The expression of some differentially expressed proteins shared by the BRVO and the recurrent and refractory groups was reversed in the post-operative group.

**Conclusions:**

Our study is the first to analyze the proteomics of recurrent, refractory, and post-operative groups treated for BRVO-ME, and may potentially provide novel therapeutic interventions for the recurrence of ME.

**Supplementary Information:**

The online version contains supplementary material available at 10.1186/s12886-024-03359-z.

## Background

Among retinal vascular disorders, branch retinal vein occlusion (BRVO) is the most frequent cause of vision impairment worldwide after diabetic retinopathy [1]. According to a 2015 epidemiological survey, the global prevalence of BRVO among people over 40 years of age was 0.64%, with the highest prevalence in Asian populations [2, 3]. BRVO can lead to various complications, among which macular edema (ME) is the most common cause of vision loss [4]. However, the exact pathogenesis of BRVO remains unknown. Currently, research on the pathological mechanism of BRVO-ME mainly focuses on two major aspects: vascular endothelial growth factor (VEGF)- and non-VEGF-related inflammatory pathways [5, 6].

VEGF is an endothelial-specific mitogen that enhances vascular permeability and angiogenesis [7]. VEGF is thought to be induced by ischemic conditions caused by retinal vein obstruction and plays an important role in the pathophysiological processes associated with BRVO-ME. Anti-VEGF therapy is the preferred treatment for BRVO-ME [8]. However, ME frequently recurs after a single dose of anti-VEGF treatment [9, 10]. Therefore, exploring the underlying causes and the subsequent physiological processes of BRVO-ME is crucial.

Recently, high-throughput technology with high accuracy and sensitivity has been developed and innovated, and has become a powerful tool for studying the pathophysiological mechanisms of organisms. Mass spectroscopy-based proteomics, which identifies and quantifies the entire proteome of a given body fluid or tissue, has been used in ophthalmic studies [11]. Protein levels in the aqueous humor (AH) both anteriorly and posteriorly, are altered in various ocular diseases [12]. Compared to vitreous fluid and plasma, the convenience and safety of AH collection increases its feasibility for future ophthalmic studies [13].

To date, few proteomic studies have been conducted on patients with BRVO-ME. In this study, four-dimensional (4D) label-free proteomics was used to conduct quantitative proteomic detection in multiple groups of patients with BRVO-ME, and differential protein screening and bioinformatics analysis were performed to explore the molecular mechanism of BRVO-ME and select potential therapeutic targets.

## Methods

### Participants

This study was approved by the Human Ethics Committee of Wuhu Eye Hospital. This prospective study was conducted in accordance with the principles of the Declaration of Helsinki. After explaining the nature and possible consequences of the study, all the participants signed informed consent forms. A total of 22 patients with BRVO-ME and 12 age-matched controls with senile cataracts treated at Wuhu Eye Hospital between September 2022 and June 2023 were selected.

The BRVO group (*n* = 14) comprised patients who had not received any treatment, including (1) a history of vitreous injection or retinal photocoagulation, (2) a history of intraocular surgery and trauma, and (3) the use of any eye drops within 3 months before sample collection. Inclusion criteria were as follows: (1) ≥ 18 years of age, (2) less than 6 months of disease duration, and (3) central retinal thickness (CRT) ≥ 250 μm.

All the 14 BRVO patients received vitreous anti-VEGF therapy, and after treatment, macular edema improved significantly with reduced CRT (Supplementary Fig. 1A, B and C). The results of long-term follow-up of these patients showed that 4 patients had a recurrence with CRT ≥ 250 μm, 7 patients did not have a recurrence of macular edema and 3 patients were lost to follow-up after the first review [14]. These four patients with recurrent macular edema were defined as the recurrence group (Supplementary Fig. 1D, E and F). To further explore the recurrence mechanism of macular edema, we selected these four recurrent patients from the 14 patients of the BRVO group and the cataract control group for secondary analysis.

In the follow-up of 14 patients of the BRVO group after treatment, we found that one patient had repeated macular edema. Although we subsequently gave the patient three times of anti-VEGF therapy and multiple retinal laser photocoagulation in the non-perfusion area under optical coherence tomography angiography (OCTA), macular edema still recurred again [15]. A total of 4 BRVO patients with macular edema again after similar treatment were collected. We defined these 4 patients as refractory BRVO and included them in the refractory group for study (Supplementary Fig. 1G, H, I, J, K, L and M).

In the 14 BRVO patients who underwent optical coherence tomography (OCT) examination one month after receiving anti-VEGF treatment, four of them still had slight macular edema. They received anti-VEGF treatment again, and the patients’ aqueous humor was collected before injection. These four patients were defined as the post-operative group. In this study, we compared the proteomic results of patients in the post-operative group with the results before the first infusion.

The exclusion criteria for all groups were as follows: (1) diabetes mellitus, (2) glaucoma, (3) trauma, (4) rubeosis iridis and hyphema, (5) vitreoretinal hemorrhage and other vitreoretinal diseases, (6) history of systemic diseases other than hypertension and (7) history of infectious diseases.

The control group was from age-matched senile patients with cataracts and a nuclear hardness rating ≥ pole II who consented to cataract surgery and gave AH samples before cataract surgery. The control group had no other systemic disease history except for hypertension, and no abnormalities were found on OCT examination.

**Fig. 1:**
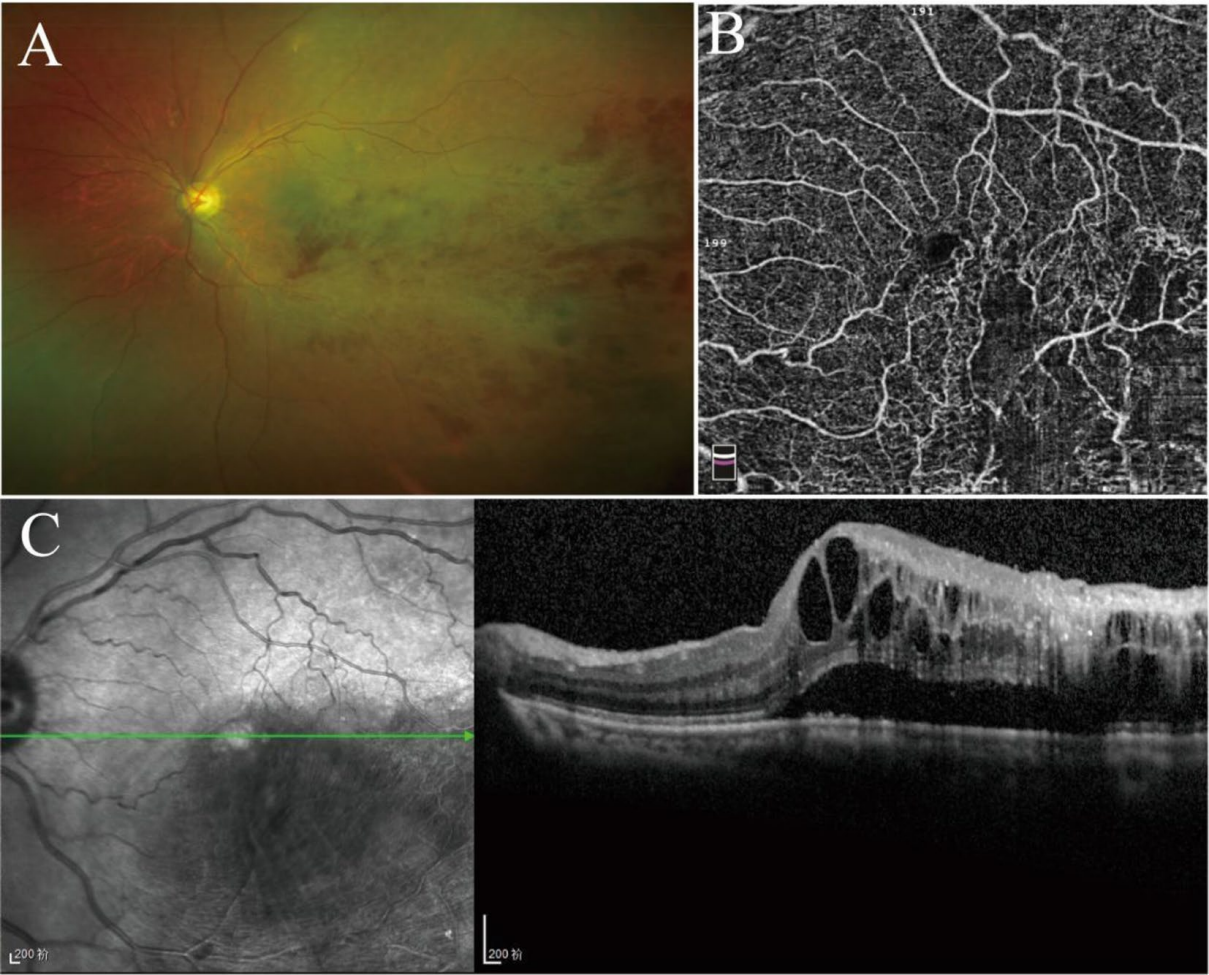
Example images taken in patients with BRVO-ME. (A) Optos ultra-widefield imaging. (B) OCTA: optical coherence tomography angiography. (C) OCT: optical coherence tomography. BRVO-ME, branch retinal vein occlusion-macular edema

A series of auxiliary examinations, including blood routine and chest routine scan, were performed before collecting the aqueous humor in all groups, and no pathological abnormalities were found. Patients with BRVO underwent comprehensive eye examinations, including visual acuity, slit-lamp biomicroscopy, OCT, OCTA and Optos ultra-widefield imaging (Fig. 1). The best corrected visual acuity was measured as the logarithm of the minimum angle of resolution. The severity of ME was measured using the caliper tool of the Heidelberg OCT software as CRT. All BRVO patients included in this study had macular edema with CRT ≥ 250 μm and received intravitreal anti-VEGF therapy.

### Sample collection

Before intravitreal injection of anti-VEGF was performed in all groups of BRVO patients, 70–90µL AH was collected under the surgical microscope through anterior chamber limbal paracentesis with a 27-gauge insulin syringe needle after disinfection and surface anesthesia. As mentioned above, AH samples from patients before cataract surgery were also collected. The AH specimens were immediately transferred to the Eppendorf tube and stored at − 80 °C for further analysis.

### Four-dimensional label-free quantitative proteome analysis of AH sample

#### Protein extraction

The sample was centrifuged at 12,000 × g for 15 min at 4 °C, and the supernatant was mixed with 1 M DTT for 1 h at 56 °C before being alkylated with sufficient iodoacetamide for 1 h at room temperature in the dark, followed by an ice bath for 2 min.

#### Protein digestion

Approximately 150 µg protein was taken from each sample. Three µg of trypsin was added to obtain a ratio of protein: enzyme = 50:1, and the samples were incubated for 14–16 h at 37℃. The enzymatically digested peptides were desalted using Waters solid-phase extraction cartridges and vacuum-dried. The dried peptide fractions were redissolved in pure water and stored at − 20 °C.

#### Reverse-phase (HpH) high-pressure liquid chromatographic (HPLC) fractionation

Equal amounts of peptides from each sample were mixed, diluted with solvent A (5% ACN, pH 9.8), and injected into a column. The peptide mixture was fractionated using a 3.5 μm 4.6 × 150 mm Agilent ZORBAX 300 Extend-C18 column on the Thermo Scientific UltiMate™ 3000 Binary Rapid Separation System. Gradient elution was performed at a flow rate of 0.3 mL/min: 5–21% solvent B (97% ACN, pH 9.8) for 38 min, 21.5–40% solvent B for 20 min, 40–90% solvent B for 2 min, 90% solvent B for 3 min, and 5% solvent B equilibrated for 10 min. The elution peaks were monitored at 214 nm, and fractions were collected every minute. The fractions were combined based on the chromatograms of the elution peaks. Ten fractions were obtained and freeze-dried.

#### Quantitative detection by Nano-LC-MS/MS

The dried peptide samples were dissolved in 0.1% formic acid and centrifuged for 10 min (20,000 × g). The supernatant was collected and injected into a self-loading C18 column (100 μm I.D., 1.8 μm column media particle size, approximately 35 cm column length). Separation was performed using the Thermo Scientific EASY-nLC™ 1200 system at a flow rate of 300 nL/min through the following effective gradient: from 0 to 103 min, 4% solvent B (98% ACN, 0.1% formic acid) increased linearly to 27%; from 103 to 111 min, solvent B increased from 27 to 40%; from 111 to 113 min, solvent B increased from 40 to 90%; and from 113 to 120 min, 90% solvent B. The separated peptides were ionized using a nano-electrospray ionization and then transferred to Orbitrap Exploris™ 480 mass spectrometer (Thermo Fisher Scientific, San Jose, CA) for data-dependent acquisition mode detection. The parameter settings were as follows: ion source voltage was 2.2 KV, the scan range of primary mass spectrometry was 350–1,500 m/z, the resolution was 60,000, the normalized automatic gain control target was 300%, and the maximum ion injection time was 20 ms. The secondary mass spectrometry fragmentation mode was higher-energy collisional dissociation. The fragmentation energy was set at 32%, resolution was set at 15,000, and dynamic exclusion time was 60 s. The starting m/z of secondary mass spectrometry was fixed at 110, the parent ion screening condition for secondary fragmentation was charge 2 + to 6+, the normalized automatic gain control target was set at the standard, and the maximum ion injection time was 22 ms.

#### Protein identification and quantification

MaxQuant (version 2.1.4.0) software was used to analyze the data-dependent acquisition label-free MS/MS data with the following settings: type: standard; enzyme: trypsin/P; maximum missed cleavages: 2; fixed modification: carbamidomethyl (C); variable modifications: oxidation (M) and acetyl (protein N-term); precursor mass tolerance: 20 ppm; fragment mass tolerance: 0.05 Da; match between runs and second peptide search was enabled. All other parameters were set to their default values. MS/MS data were searched for protein sequences downloaded from the UniProt database. The FDR threshold was set at 1% for both peptide spectrum matching and protein levels. Proteins from the contaminants or reverse were removed.

### Statistical analysis

Statistical analyses were performed using R software (version 4.0.0). The raw protein intensity was normalized via the “medium” method. Hierarchical clustering was performed using the heatmap package. Principal component analysis (PCA) was performed using the meta-package. A t-test was used for statistical differential analysis, and a cut-off of adjusted *p*-value < 0.05 and fold change (FC) ≥ 1.2 was used to select statistically differential expressed proteins [16]. Hypergeometric-based enrichment analysis using the Kyoto Encyclopedia of Genes and Genomes (KEGG) pathway, Gene Ontology (GO), and Reactome pathways was performed to annotate the protein sequences individually. Subcellular localization analysis was performed using WoLF PSORT. Transcription factor annotation was based on AnimalTFDB. GraphPad Prism (version 9.5.0) was used to perform Student’s t-tests to examine the findings statistically.

## Results

### Patient characteristics

A total of 34 patients were included in this study, including four groups of patients with BRVO-ME and 12 age-matched cataract controls without retinal pathology. The characteristics of the patients are shown in Table 1. No significant difference was observed in age among all groups (*p* > 0.05).

**Table 1:** Characteristics of the patients

Items	BRVO (*n* = 14)	Control(*n* = 12)	P Value	Recurrence (*n* = 4)	Control(*n* = 12)	P Value	Refractoriness (*n* = 4)	Control(*n* = 12)	P Value
Age (years)	64.00 ± 11.50*	66.25 ± 4.77*	0.53	64.00 ± 1.63*	66.25 ± 4.77*	0.38	60.75 ± 9.74*	66.25 ± 4.77*	0.15
Sex (M/F)	4/11	1/11		1/3	1/11		0/4	1/11	
Size of macular edema (um)	644.6 ± 218.5*			463.8 ± 171. 2*			525.8 ± 234.3*		
BCVA	0.19 ± 0.22*			0.26 ± 0.11*			0.24 ± 0.18*		
Hypertension (yes/no)	3/11	3/9		0/4	3/9		1/4	3/9	

*Data are expressed as mean ± standard deviation. BRVO, branch retinal vein occlusion; BCVA, best-corrected visual acuity

### Proteomic analysis between the BRVO and control groups

**Fig. 2:**
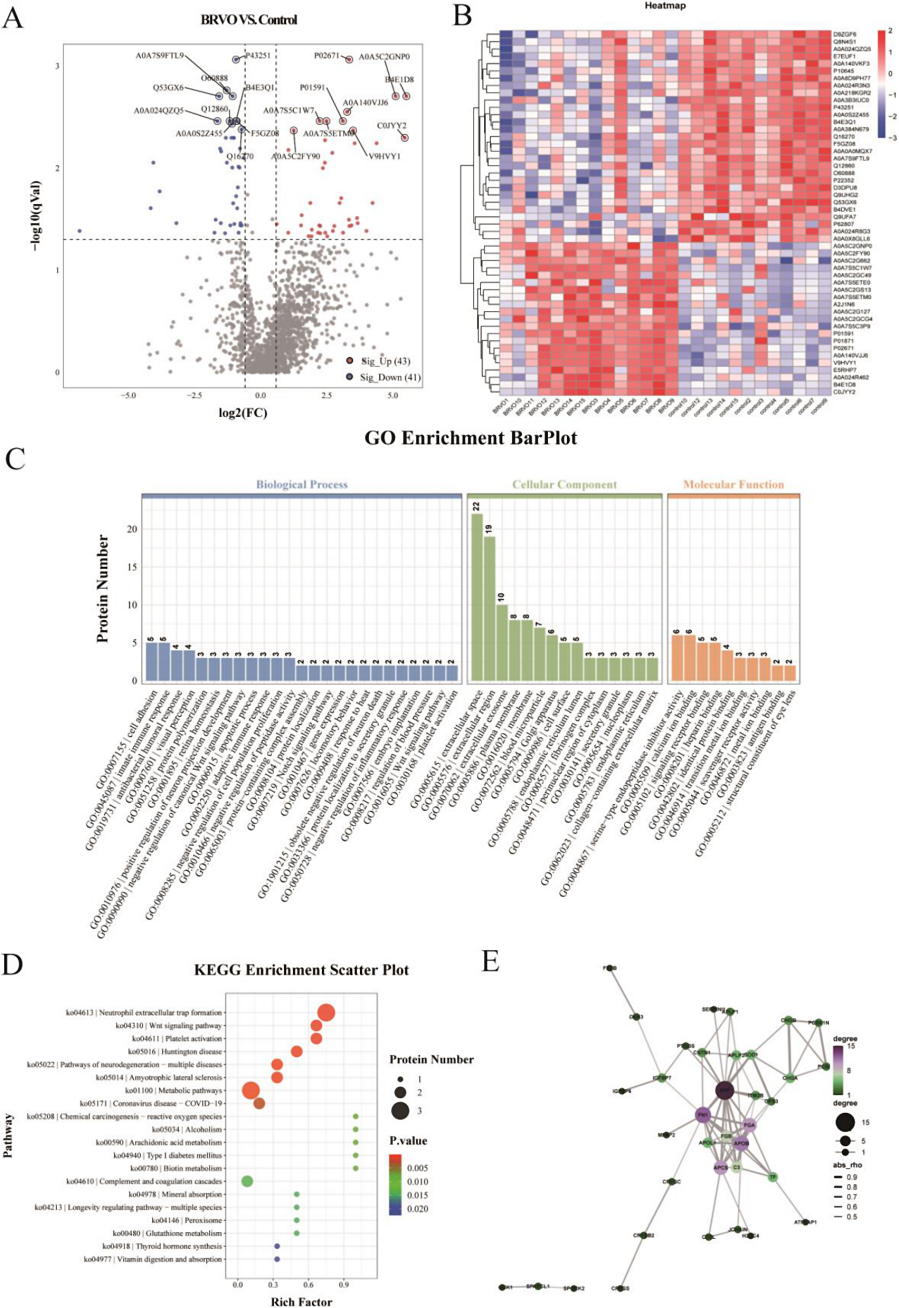
Proteomic analysis of the BRVO and control groups. (A) Volcano plots. (B) Heat map representation of the top 50 differential proteins. Each line in the heat map represented a protein. The deeper the red color, the higher its content in the tested sample; similarly, the deeper the blue color, the lower its content in the tested sample. (C) Gene Ontology (GO) enrichment bar plot. (D) KEGG enrichment bubble diagram. (E) PPI sub-network obtained by Cytoscape in BRVO compared to controls. The size of the bubble was related to the connectivity. The thickness of the line indicated the strength of the correlation. BRVO, branch retinal vein occlusion; KEGG, Kyoto Encyclopedia of Genes and Genomes; PPI, protein-protein interaction

The number of proteins detected in all samples is shown in Supplementary Fig. 2. Approximately 84 proteins exhibited significant differential changes between the BRVO and control groups (FC ≥ 1.2 and adjusted *p*-value < 0.05). Compared to the control group, 43 proteins were upregulated, and 41 proteins were downregulated in the BRVO group (Fig. 2A). A heatmap of the differentially expressed proteins between the two groups is shown in Fig. 2B. Subcellular localization statistics were analyzed for different proteins in each comparison group. The top five subcellular locations with the highest number of proteins were extracellular (36), cytosolic (20), nuclear (16), plasma membrane (5) and cyto-nuclear (4) regions (Supplementary Fig. 3A).

In the Gene Ontology (GO) enrichment analysis, the biological processes mainly included cell adhesion, visual perception, retina homeostasis and platelet activation (Fig. 2C). Through significant enrichment of pathways, the most important pathways involved in differentially expressed proteins were identified (Fig. 2D). Differences in proteins mainly involved the Wnt signaling pathway, platelet activation, metabolic pathways and complement and coagulation cascades. InterPro enrichment identified the domain entries that were significantly enriched. The differences in InterPro enrichment can be seen in Supplementary Fig. 3B. Enrichment analysis of disease ontology (DO) annotations was performed to obtain possible links between proteins and diseases. The first 20 items with the smallest *p*-values in the DO enrichment analysis were screened (Supplementary Fig. 3C). The top 20 DO entries with the adjusted smallest *p*-values were Alzheimer’s disease, cataracts, obesity and hypertension. Possibly related or interacting proteins were found using protein–protein interaction (PPI) in the search tool for retrieval of interacting genes (STRING) protein interaction database. PPI analysis in the two groups revealed 10 major clusters of differential molecules, with seven upregulated (FN1, APOB, FGA, APCS, C3, FGB and APOL1) and three downregulated (Amyloid Precursor Protein, APLP2 and CHGA) (Fig. 2E).

### Proteomic analysis among the recurrent, refractory, and control groups

**Fig. 3:**
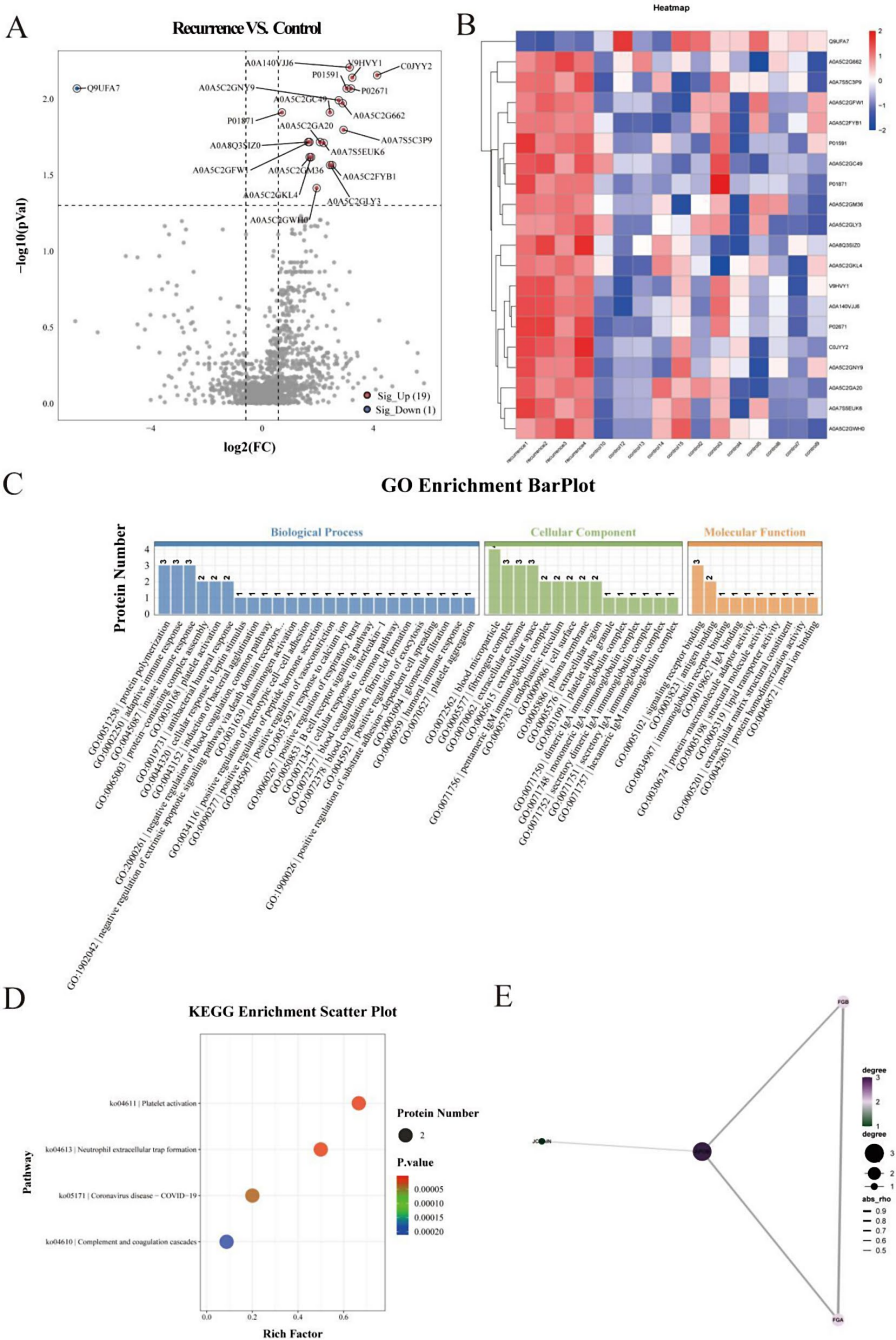
Proteomic analysis of the recurrent and control groups. (A) Volcano plots. (B) Heat map representation of the 20 differential proteins. (C) Gene Ontology (GO) enrichment bar plot. (D) KEGG enrichment bubble diagram. (E) PPI sub-network obtained using Cytoscape. KEGG, Kyoto Encyclopedia of Genes and Genomes; PPI, protein-protein interaction

**Fig. 4:**
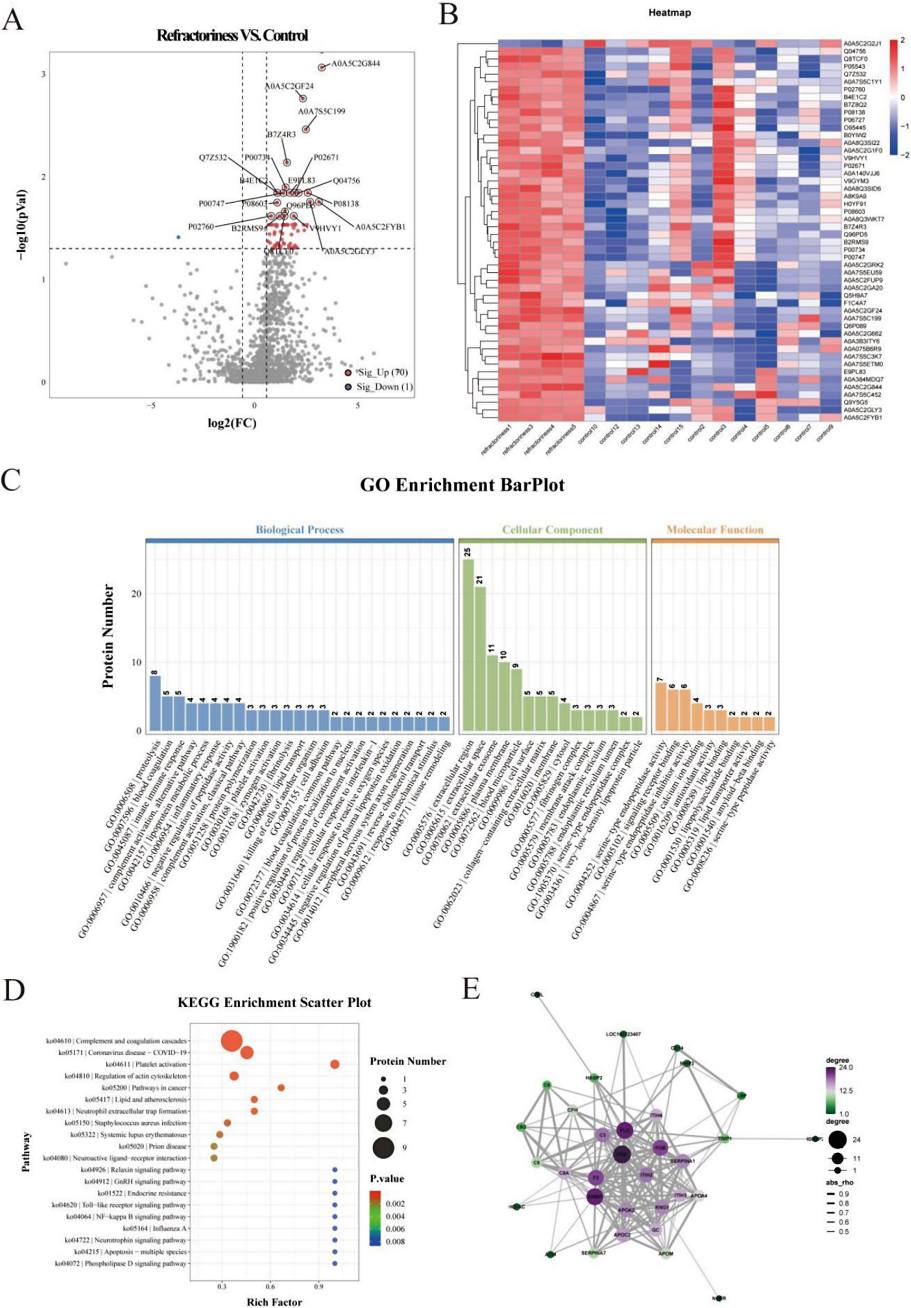
Proteomic analysis of the refractory and control groups. (A) Volcano plots. (B) Heat map representation of the top 50 differential proteins. (C) Gene Ontology (GO) enrichment bar plot. (D) KEGG enrichment bubble diagram. (E) PPI sub-network obtained using Cytoscape. KEGG, Kyoto Encyclopedia of Genes and Genomes; PPI, protein-protein interaction

Compared to the control group, 19 proteins were upregulated and one protein was downregulated in the recurrent group (Fig. 3A); however, 70 proteins were upregulated, and one was downregulated in the refractory group (Fig. 4A). Protein cluster analysis results are shown in Figs. 3B and 4B. Subcellular localization statistics are shown in Supplementary Fig. 3D and G.

Gene Ontology (GO) enrichment analysis revealed biological processes, including platelet activation, blood coagulation, and platelet aggregation in the recurrent and control groups. However, blood coagulation, complement activation, lipoprotein metabolic processes, inflammatory response, and platelet activation were observed in the refractory and control groups (Fig. 3 C). KEGG pathway analysis showed that proteins were differentially expressed among the three groups, primarily in the complement and coagulation cascades and platelet activation (Figs. 3D and 4D). The InterPro and DO enrichment analyses are shown in Supplementary Fig. 3E, F, H and I.

The PPI analysis results showed only four major clusters of differential molecules, with four upregulated (APOB, FGA, FGB and JCHAIN) between the recurrent and control groups (Fig. 3E). In contrast, 10 major clusters of differential molecules, with 10 upregulated (FGA, AMBP, PLG, Factor II, FGB, C3, ITIH2, APOA2, KNG1 and SERPINA1), were observed between the refractory and control groups (Fig. 4E).

### Selection of potential biomarkers for the recurrence of macular edema

**Fig. 5:**
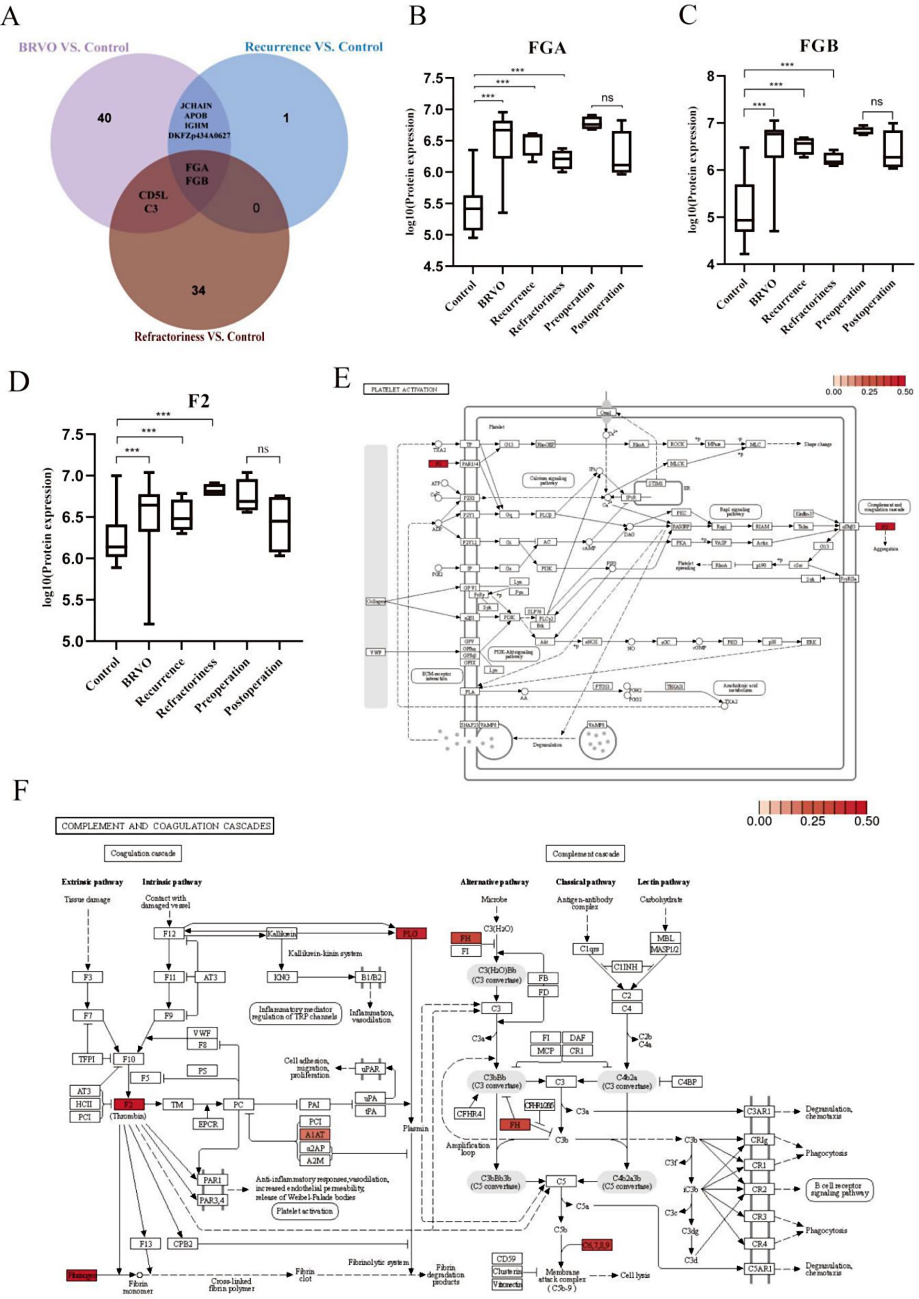
Comprehensive analysis of proteomic data from multiple groups. (A) Venn diagram analysis of protein interaction among BRVO, recurrence, refractoriness and control. (B, C and D) The up- or down-regulation of FGA, FGB, and F2 protein expression in every two groups. (E) platelet activation in refractory and control groups. (F) complement and coagulation cascade in refractory and control groups. FGA, fibrinogen alpha chain; FGB, fibrinogen beta chain; F2, coagulation factor II.

Venn chart analysis was performed on the PPI results among the BRVO, recurrent, refractory, and control groups (Fig. 5A). We found that FGA and FGB were common among all groups. Compared to the control group, the expression of FGA, FGB and factor II (F2) proteins among the BRVO, recurrent, and refractory groups was significantly upregulated, and their expressions were downregulated after anti-VEGF treatment (Fig. 5B, C and D). Comparative analysis of the KEGG pathway showed that the complement and coagulation cascades and platelet activation were involved in all groups; three proteins were enriched in the platelet activation pathway and eight proteins in the complement and coagulation cascade pathway in the refractory group, and these proteins showed varying degrees of upregulation (Fig. 5E and F).

## Discussion

BRVO is an ocular disease characterized by retinal vascular abnormalities. BRVO occurs when a branch of the central vein is blocked [17], and the retina develops ischemia upstream of the occlusion, resulting in changes in several inflammatory proteins together with increased VEGF levels. This eventually leads to ME, which carries the risk of blindness or severe vision loss [18]. The AH plays an important role in maintaining homeostasis in the eyes, and analysis of the AH can provide new insights into our understanding of the pathophysiology of retinal diseases [13]. This study aimed to analyze the AH protein profiles of patients with BRVO and identify potential biomarkers related to disease pathophysiology. The 4D label-free method used in this study has the advantages of low cost, good reproducibility, fewer sample-processing procedures, and fewer sample quantity requirements [19].

To date, few studies exist on the proteomic analysis of the AH in patients with BRVO. Yao et al. [12] conducted a proteomic study on AH samples from six patients with BRVO and secondary ME and six control patients with cataracts using two-dimensional electrophoresis combined with mass spectrometry and bioinformatics analysis. A total of 49 protein sites were identified. Several of these proteins are involved in oxidative stress, angiogenesis, and collagen synthesis. Cehofski et al. [20] analyzed AH samples from 19 untreated patients with BRVO-ME and 18 age-matched controls with cataracts using label-free quantitative proteomic analysis. Statistically significant changes were observed in the expression of 52 proteins. Several proteins, including fibrinogen chains, apolipoprotein C-III, and complement factors, are associated with CRT. Fibronectin levels are closely related to the BRVO severity.

The most significant finding of this study was the involvement of the complement and coagulation cascades in BRVO progression. In thrombosis and inflammatory diseases, complement and coagulation cascades are functional pathways commonly enriched in bioinformatics studies. Low levels of complement and coagulation cascades in the eye contribute to the homeostasis and integrity of the retina [21]. Various pathophysiological changes activate the complement and clotting pathways, including increased vascular permeability [22] and inflammation [23].

In our study, we found that the platelet activation pathways were also significantly enriched in all groups. Platelet activation may be responsible for the recurrence of ME. Platelets are important effector cells involved in hemostasis, coagulation, and thrombosis. However, excessive platelet activation can lead to thrombosis and coagulopathy. The activated platelets release lysosomes, A granules, and dense granules. These elements regulate several physiological processes, such as hemostasis, coagulation, inflammation, vasoconstriction, and angiogenesis [24, 25].

FGA and FGB are involved in platelet activation and complement and coagulation cascades. The expression levels of FGA and FGB were significantly upregulated in the BRVO, recurrent, and refractory groups. The two polypeptide chains constituting fibrinogen are FGA and FGB. An increase in FGA and FGB, two essential proteins at the end of the extrinsic coagulation (EC) pathway, shows that this pathway has been engaged, and the blood has become hypercoagulable [26]. The FGA is a fibrinogen subunit consisting of 866 amino acids with a molecular weight of approximately 95 kDa [27]. FGA are plasma glycoproteins that affect the coagulation cascade [28]. The large plasma glycoprotein FGB exists as a dimer that is converted to a fibrin dimer during prolonged contact with thrombin [29]. In the coagulation pathway, FGB plays a significant role in controlling endothelial function and platelet aggregation [30].

Another protein involved in platelet activation and the complement and coagulation cascades in both the recurrent and refractory groups was F2. The expression of coagulation F2 was not significantly upregulated in the BRVO group; however, it was significantly upregulated in the recurrent and refractory groups. Therefore, we speculated that F2 is involved in the recurrence of ME. F2 is a multidomain glycoprotein vital for life and a primary target for anticoagulant therapy [31]. Prothrombin, encoded by F2, is necessary for blood clot development. Prothrombin is transformed into thrombin, which converts fibrinogen into fibrin when blood vessels are damaged [32]. Both fibrinogen and fibrin directly affect leukocyte migration and inflammatory responses of endothelial cells and leukocytes [33].

Notably, we found some down-regulated differential proteins such as Amyloid Precursor Protein (APP) and Transthyretin (TTR) in the BRVO group compared to the control group. APP is a widely expressed transmembrane protein that is thought to play a function in the development of Alzheimer’s disease [34]. APP is a Kunitz-type protease inhibitor that activates coagulation factor XII, influencing the hemostasis and temporal stability of the thrombus [35]. TTR is a homotetramer carrier protein secreted by human retinal pigment epithelial cells in ocular tissue [36]. TTR can inhibit retinal vascular proliferation in Diabetic Retinopathy [37]. In experimental BRVO, bevacizumab treatment led to enhanced TTR [38].

This study had some limitations. First, the sample size was small. In the future, a larger sample size will reduce variation in the results. Second, because the proteomic detection of low-abundance proteins is affected by many factors, some relevant low-abundance proteins, such as VEGF, interleukin-6, and interleukin-8, were not identified in our proteomic analysis, and these will be considered when verifying our findings in the future. Finally, we did not verify the results of this study, which we plan to do in subsequent studies.

## Conclusions

In summary, ME recurrence in branch retinal veins was thoroughly examined in this study. This study is the first to analyze the proteomics of recurrent, refractory, and post-operative groups with BRVO-ME. We found that FGA, FGB, complement and coagulation cascades, and platelet activation are involved in BRVO development. FGA, FGB, and F2 may be responsible for ME recurrence, leading to the development of novel therapeutic interventions for ME recurrence.

### Electronic supplementary material

Below is the link to the electronic supplementary material.

Supplementary Fig. 1. The representative OCT images of BRVO, recurrence and refractoriness groups. Supplementary Fig. 2. The number of proteins detected in all samples. Supplementary Fig. 3. Subcellular localization, InterPro enrichment, and DO enrichment among BRVO, recurrent, refractory and control groups.

## Data Availability

Data is provided within the manuscript or supplementary information files.

## Abbreviations

BRVO: Branch retinal vein occlusion
ME: Macular edema
VEGF: Vascular endothelial growth factor
AH: Aqueous humor
4D: Four-dimensional
OCT: Optical coherence tomography
OCTA: OCT angiography
HPLC: High-pressure liquid chromatography
PCA: Principal component analysis
KEGG: Kyoto Encyclopedia of Genes and Genomes
GO: Gene Ontology
STRING: Search tool for retrieval of interacting genes
PPI: Protein–protein interaction
