# Supplementary material for: Recurrence of macular edema in patients with branch retinal vein occlusion: a proteomic study
Source: BMC Ophthalmol. 2024 Feb 22;24:82. doi: 10.1186/s12886-024-03359-z (PMC10882909; doi:10.1186/s12886-024-03359-z)
Supplement: Supplementary file 1 — Supplementary Fig. 1. The representative OCT images of BRVO, recurrence and refractoriness groups. Supplementary Fig. 2. The number of proteins detected in all samples. Supplementary Fig. 3. Subcellular localization, InterPro enrichment, and DO enrichment among BRVO, recurrent, refractory and control groups. [file 12886_2024_3359_MOESM1_ESM.docx]

Supplementary Fig. 1


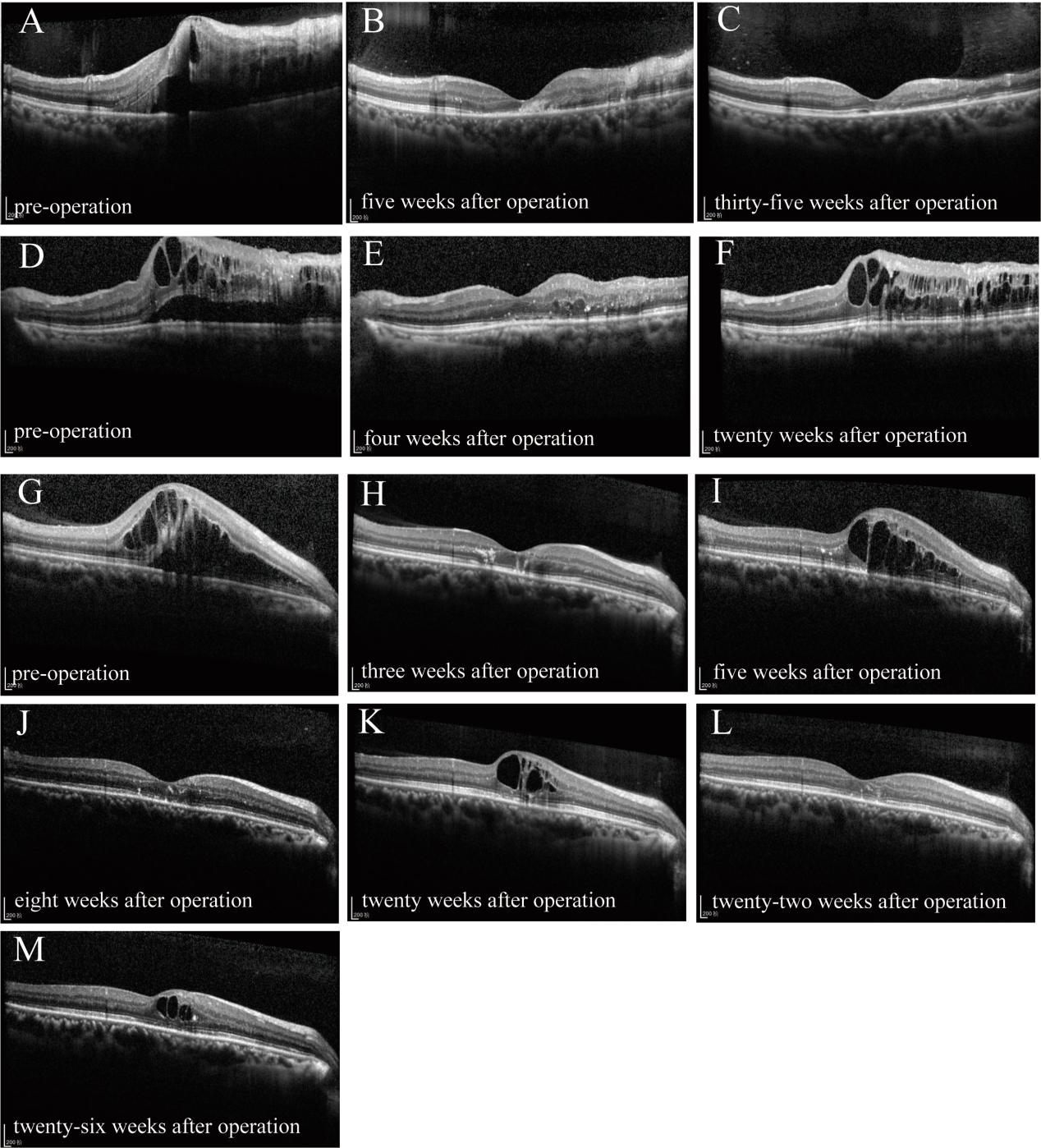


The representative OCT images of BRVO, recurrence and refractoriness groups.(A, B and C) The OCT images of BRVO patient before injection as well as after operation at five and thirty-five weeks. (D, E and F) Dynamic observation of the recurrent individual’s OCT images. (G, H, I, J, K, L and M) A long-term OCT examination of refractory patient presented repeated macular edema. OCT, optical coherence tomography; BRVO, branch retinal vein occlusion;

Supplementary Fig. 2


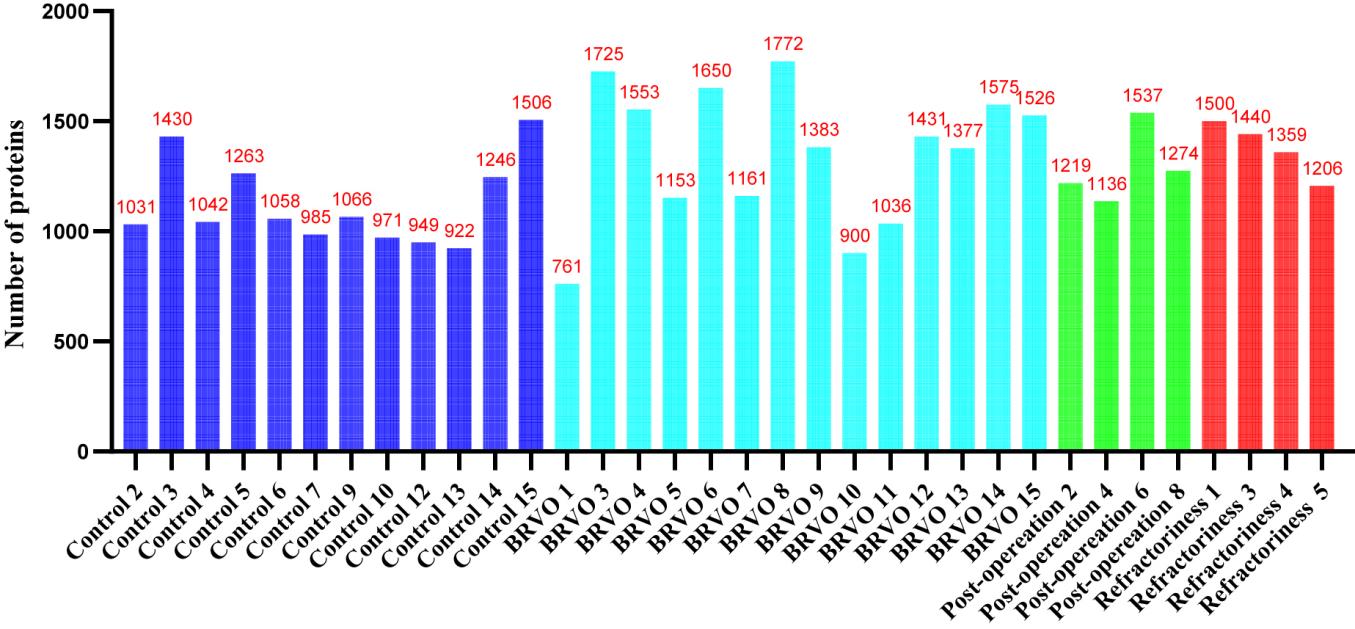


The number of proteins detected in all samples.

Supplementary Fig. 3


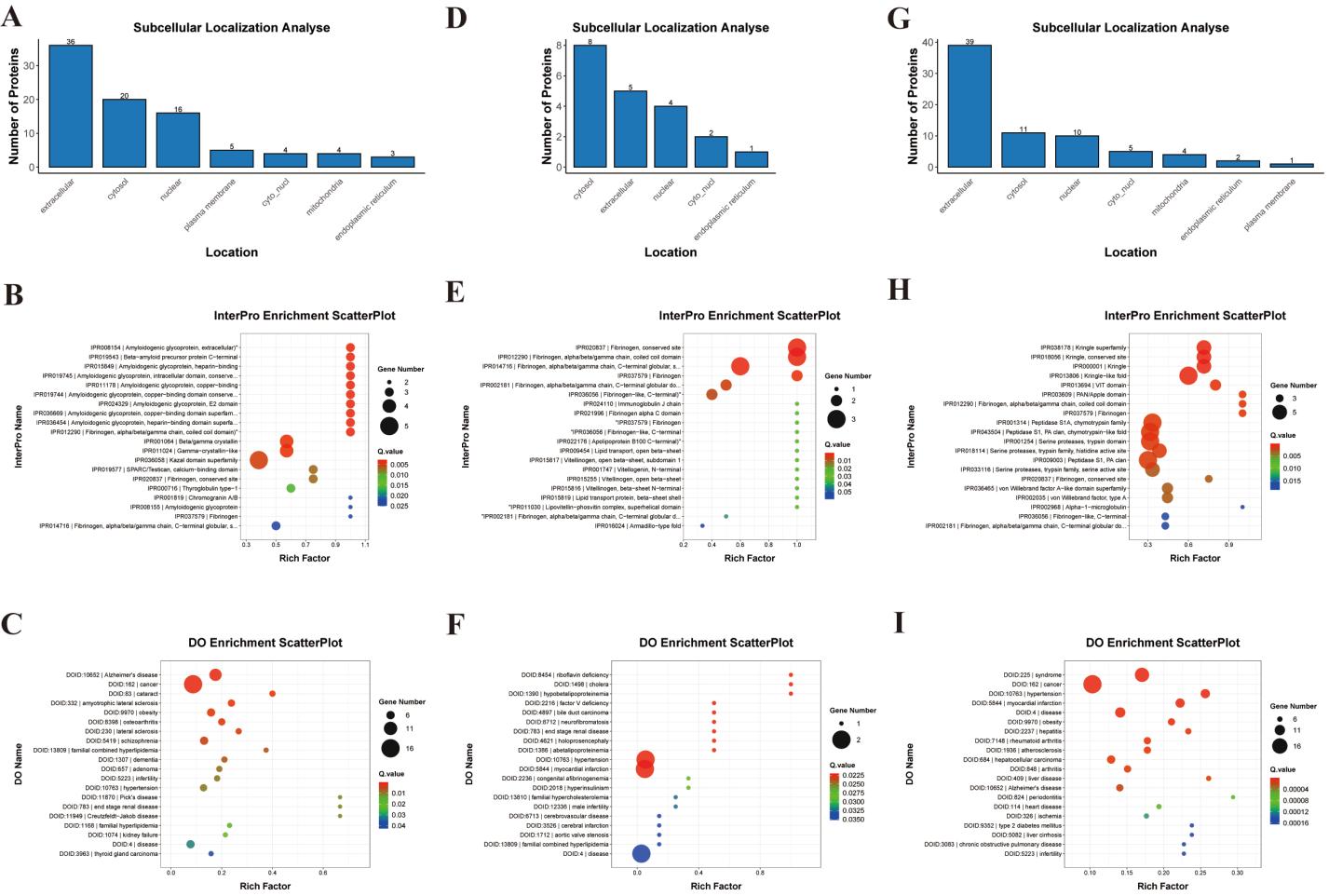


Subcellular localization, InterPro enrichment, and DO enrichment among BRVO, recurrent, refractory and control groups.(A, D and G) Subcellular localization statistics. (B, E and H) InterPro enrichment bar plot. (C, F and I) DO enrichment bar plot. BRVO, branch retinal vein occlusion; DO, Disease Ontology.
